# Comparative Analysis and Safety Evaluation of Shield Segment Structure Model under Surcharge Loading

**DOI:** 10.3390/ma16206806

**Published:** 2023-10-22

**Authors:** Xiaofeng Liu, Yan Jiang, Xiaolong Li, Quansheng Zang, Jinchao Yue

**Affiliations:** School of Water Conservancy and Transportation, Zhengzhou University, 100 Science Avenue, Zhengzhou 450001, China; lxf18728635637@163.com (X.L.); 13073792750@163.com (Y.J.); wennuandeshang@hotmail.com (X.L.); qszang1991@zzu.edu.cn (Q.Z.)

**Keywords:** shield segment, surcharge loading, shell–spring model, beam–spring model, safety evaluation

## Abstract

In shield tunneling projects, the selection of an accurate model to calculate the mechanical response of segment structure plays a crucial role in the design and cost of the project. The shell–spring and beam–spring models are two widely used methods for this purpose. However, it is still not clear how accurate and different these models are in calculation results under surcharge load. Therefore, to accurately calculate the internal forces and deformation of the segment structure and clarify the difference between the two models’ results, the shell–spring and beam–spring models were established based on a subway shield tunnel project in Zhengzhou city. The reliability of the models was verified by comparing and analyzing the differences in deformation results between the models and field measurements. Furthermore, the safety of the segment structure was evaluated according to the ultimate bearing capacity of the normal section. The results declare that: (1) In the shell–spring model, the internal force gradually reduces from the edges towards the center of the segment width, and the shield segment exhibits a prominent non-plane strain state. (2) The internal force of the beam–spring model is larger than that of the shell–spring model. The axial force difference between the two models is relatively small; meanwhile, there is a larger disparity in the bending moment. However, with an increase in surcharge loading, the discrepancy in internal forces between the two models gradually decreases. (3) The calculation results of the shell–spring model are close to the field-measured values and the shield tunnel model test values, which verifies the accuracy and reliability of the shell–spring model. Therefore, it is more reasonable to use the shell–spring model to calculate the mechanical response of the segment structure. (4) With an increase in surcharge loading, the safety of the shield tunnel decreases gradually. Therefore, surcharge loading above the shield tunnel should be reasonably controlled to meet the requirements of the normal use of the shield segment. This manuscript aims to provide a reference for the future design and optimization of the shield tunnels’ lining structure.

## 1. Introduction

In recent years, urban rail transit has become an important component of the public transportation systems in many large cities. Shield tunnels, as the main structural form of urban rail transit sections, are crucial for the safe operation of subway systems. However, with the increasing mileage of urban rail transit and rapid urban development, the phenomenon of soil abandonment caused by nearby construction activities has become increasingly common. Surcharge loading imposes additional loads on the shield tunnel segments, increasing their deformation and internal forces, and thereby reducing the structural safety of shield tunnel segments [1]. Accidents resulting from tunnel structure damage caused by surcharge loading have occurred frequently, posing a serious threat to the safety of shield tunnel structures [2]. Therefore, it is very important to study the mechanical response characteristics of the shield segment under surcharge loading and evaluate its safety performance to maintain the normal operation of the shield tunnel [3]. Currently, scholars have conducted a series of studies on the mechanical response and deformation mechanism of shield tunnel lining structures under surcharge loading using various research methods, including theoretical analysis [4,5,6], numerical simulation [7,8,9], field measurements [10,11,12], and model tests [13,14,15,16]. Among these methods, numerical models have been favored by researchers due to their ability to conveniently calculate the mechanical response characteristics of shield tunnel structures under various loading conditions.

To improve the accuracy of numerical models for shield tunnel segments, numerous scholars have extensively investigated the establishment of numerical models that better reflect the actual behavior of shield tunnel segments. Feng et al. [17] idealized the shield tunnel as an infinite beam on the three-parameter Kerr model and established the vertical force balance equation of the tunnel element. Chaipanna et al. [18] proposed a nonlinear foundation spring model that considered yield pressure and applied it to a numerical analysis. Chen et al. [19] established a three-dimensional solid element model to investigate the structural behavior of the segmental ring. Jiang et al. [20] used the modified conventional method and beam–spring method to compare and analyze the internal force and deformation of the shield segment. Arnau et al. [21] established a two-dimensional plane stress model and a three-dimensional shell element model and calculated and analyzed the mechanical response characteristics of shield segments. Rashiddel et al. [22] used the beam–spring method and solid–interface method to analyze the mechanical response of the shield tunnel. Yang et al. [23] proposed an improved three-dimensional solid spring model and a simple two-dimensional beam–spring model to consider the influence of segment joints. Huang et al. [24] established a calculation algorithm for the effective ratio of transverse bending stiffness. Zhu et al. [25] proposed that the beam–spring model can be divided into the beam–spring continuous model and the beam–spring discontinuous model according to the different treatments of the node displacement of adjacent segments at the joint position. Zhu et al. [26] used the shell–spring model and the beam–spring model to compare and analyze the through-seam and staggered-seam assembly of lining segments. Pham et al. [27] clarified the influence and mechanism of tangential stratum–lining interaction on segment behavior. Sun et al. [28] proposed a segment structure model based on the shell–spring model and improved the simulation method of segment joints. Wen et al. [29] established the finite element model of the beam–spring method and analyzed the sensitivity of the corresponding internal force curve by changing the spring stiffness value. Liu et al. [30] established the shell–joint model of the segment indirect joint and believed that the calculated value of the bending moment was smaller than that of the segment joint; meanwhile, the axial force value was closer. Kim et al. [31] established a two-dimensional tunnel analysis model to replace the beam–spring model and proposed the application of the model in a tunnel monitoring system. Saito et al. [32] proposed a new numerical analysis method that combines the beam–spring model and discrete element method. Li et al. [33] used a three-dimensional finite element method and a set of full-scale tests to study the performance of cast iron tunnel segments used in underground tunnels. Zhou et al. [34] proposed a shell–matrix–spring model for the calculation and analysis of segment structure and proved its applicability. Guan et al. [35] established a homogeneous ring model and a shell–spring model, and the bending stiffness effective ratio and bending moment transfer ratio parameters of the two models were compared and analyzed.

The above scholars have introduced a variety of internal force calculation methods related to segment structure. However, there is no unified calculation model for the safety assessment of shield segment structure. Commonly used methods include the customary method, modified customary method, multi-hinge ring method, beam–spring model method, and shell–spring model method [36,37,38]. Among these, the beam–spring model and the shell–spring model have the advantages of simple modeling and high computational efficiency, and can better simulate the stress state of the shield segment; therefore, they are widely used. However, the results calculated using these two models are different. At present, the difference between the calculation results of the beam–spring model and the shell–spring model is not clear enough. Especially under different surcharge loading, there are relatively few studies on the difference between the calculation results of the beam–spring model and the shell–spring model.

**Figure 1:**
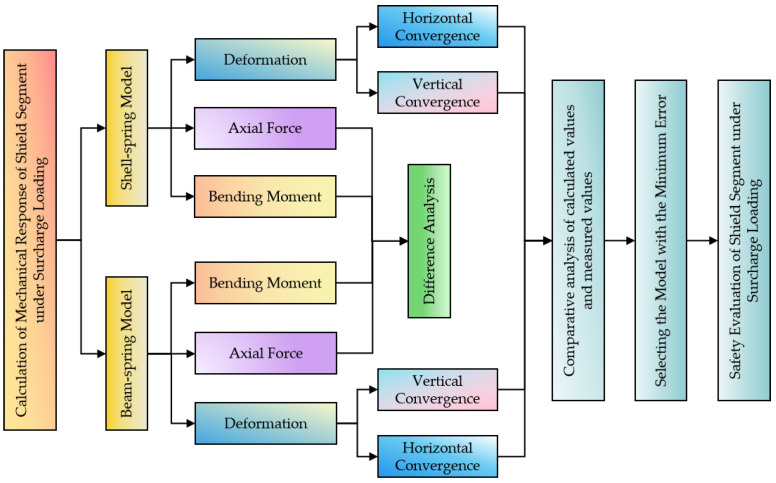
The research technical route flowchart.

To accurately calculate the internal force and deformation of the segment structure under different surcharge loadings and clarify the difference between the calculation results of the two models, the shell–spring and beam–spring models are established, respectively, based on a subway shield tunnel project in Zhengzhou city. The difference in the internal force calculation results of the segment under the two models is compared and analyzed. By comparing and analyzing the difference between the deformation calculation results of the two models and the field-measured values, the reliability of the model is verified [39,40,41]. Finally, the model that is closer to the measured data is selected to calculate the ultimate bearing capacity of the shield segment and evaluate its safety. The research technical route of this paper is shown in Figure 1.

## 2. Project Overview

### 2.1. Engineering Geological Conditions

**Figure 2:**
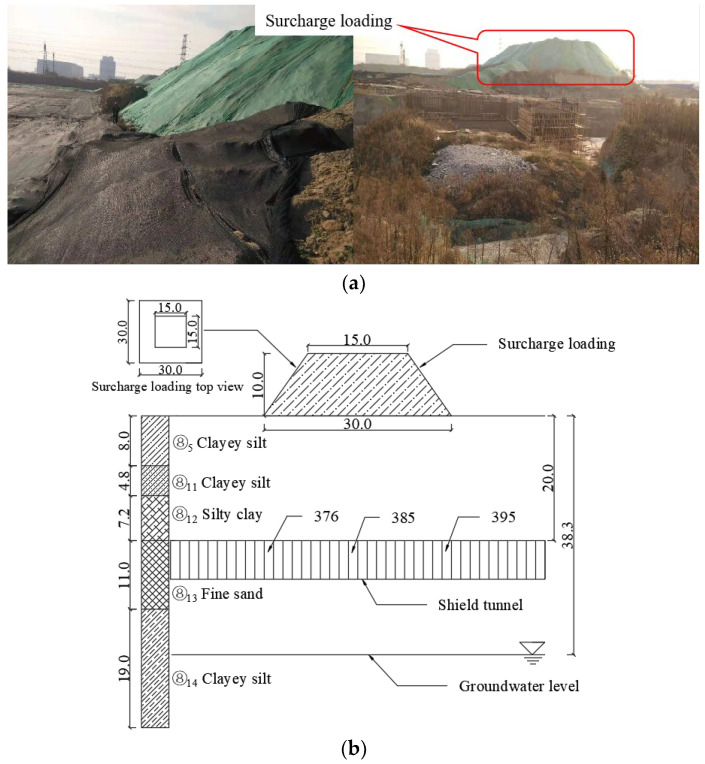
Site condition of shield tunnel: (**a**) surcharge loading and (**b**) stratum structure diagram (unit: m).

The shield tunnel is located in the alluvial plain of the Yellow River in Zhengzhou city. The soil layer in this area is mainly silt, the foundation resistance coefficient is 10 MPa/m, and the stability of the surrounding rock structure is poor. A diagram of surcharge loading and stratum structure above 376~395 ring segments is shown in Figure 2. Above the shield tunnel, there is a pile of soil with a bottom of 30 m × 30 m, an upper of 15 m × 15 m, and a 10 m high. The strata from top to bottom are ⑧_5_ clayey silt, ⑧_11_ clayey silt, ⑧_12_ silty clay, ⑧_13_ fine sand, and ⑧_14_ clayey silt. The buried depth of the tunnel is 20 m, the stable water level is 38.3 m, and the groundwater is below the bottom of the tunnel. The physical and mechanical parameters of each soil layer are shown in Table 1.

### 2.2. Parameters of Lining Structure

**Figure 3:**
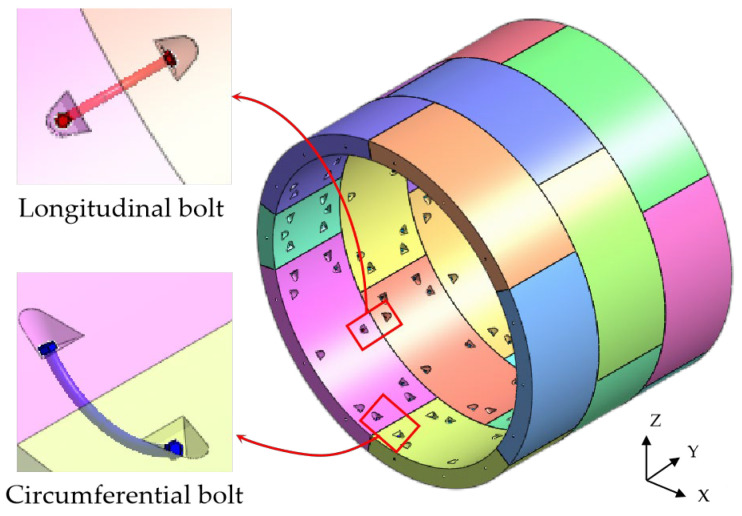
A schematic diagram of the shield lining structure.

**Figure 4:**
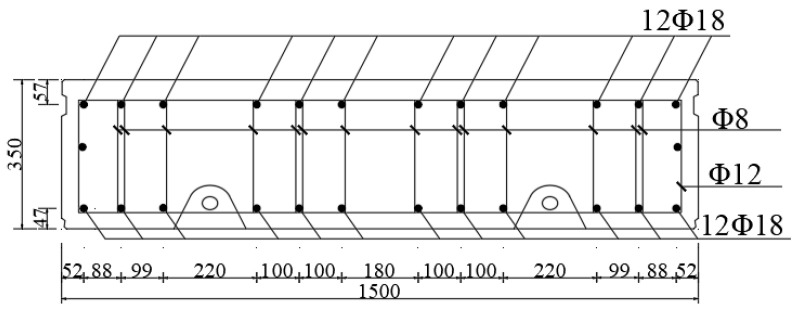
A schematic diagram of a normal segment section with a reinforced structure.

The schematic diagram of the shield lining structure is shown in Figure 3. X is the horizontal direction of the lining ring, Y is the longitudinal direction of the lining ring, and Z is the vertical direction of the lining ring. The outer diameter of the shield lining is 6.2 m, the inner diameter is 5.5 m, and the ring width is 1.5 m. The joints of the lining ring are connected by bending bolts, including 16 longitudinal joint bolts (M30) and 12 circumferential joint bolts (M30) [42,43,44,45]. The tunnel segments are assembled using staggered joints, and the angle of staggered joints between longitudinal segments is 45°. The normal section reinforcement structure of the shield segment is shown in Figure 4. The concrete grade of the segment is C50, the diameter of the main reinforcement is 18 mm, the diameter of the stirrup is 8 mm, and the steel bar model is HRB400 [27,28].

## 3. Calculation Equations of Load and Ultimate Bearing Capacity of Lining Segment

### 3.1. Load Calculation of Lining Segment

**Figure 5:**
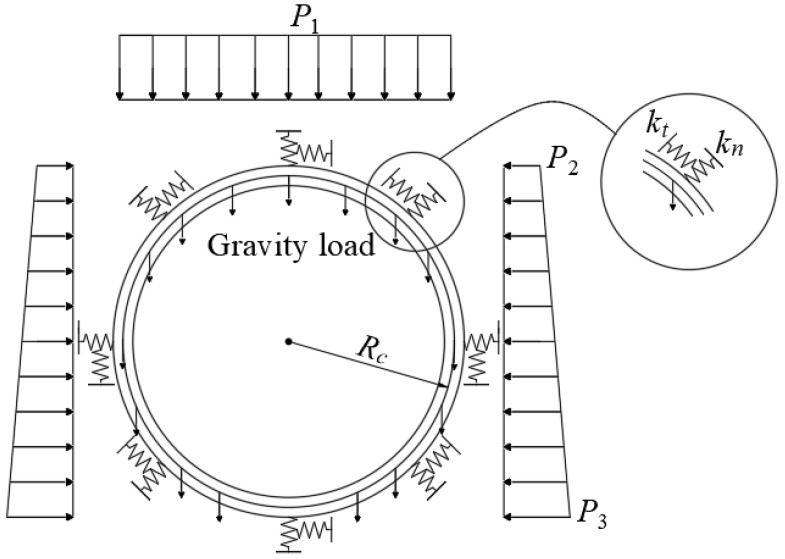
Schematic diagram of the shield segment load structure.

In this paper, the mechanical response of the shield segment is studied using the load-structure method. The interaction between the shield segment and stratum is simulated using the “Winkler” foundation spring [46]. The load of the shield segment in the stratum is shown in Figure 5, where *k_n_* is the radial spring stiffness and *k_t_* is the tangential spring stiffness. The vertical earth pressure and lateral earth pressure of the shield segment in the stratum are calculated using Terzaghi’s tunnel loosening earth pressure formulas [47].

#### 3.1.1. Vertical Earth Pressure

(1) $$P_{1}=\frac{B\gamma -c}{K\tan\varphi }(1-e^{-K\tan\varphi \frac{H}{B}})+qe^{-K\tan\varphi \frac{H}{B}}$$

(2) $$B=2R_{c}+2R_{c}\tan(45^{{}^{\circ}}-\frac{\varphi }{2})$$

where *P*_1_ is the vertical earth pressure (kPa), *R_c_* is the calculated radius of the tunnel (m), *H* is the tunnel depth (m), *γ* is the average formation weight (kN/m^3^), *C* is the average cohesion (kPa), *φ* is the average internal friction angle (°), *K* is the lateral pressure coefficient, and *q* is surcharge loading (kPa).

#### 3.1.2. Lateral Earth Pressure

(3) $$P_{2}=P_{1}\tan^{2}(45^{\circ }-\frac{\varphi }{2})$$

(4) $$P_{3}=P_{2}+2\gamma R_{c}\tan^{2}(45^{\circ }-\frac{\varphi }{2})$$

where *P*_2_ is the lateral earth pressure at the top of the tunnel (kPa) and *P*_3_ is the lateral earth pressure at the bottom of the tunnel (kPa).

### 3.2. Calculation of the Normal Section Ultimate Bearing Capacity of Lining Segment

#### 3.2.1. Determining Large or Small Eccentric Compression

Limit eccentricity equation:

(5) $$e_{0b}=\frac{f_{c}b\xi_{b}h_{0}\left(h-\xi_{b}h_{0}\right)+f_{y}{}^{\prime }A_{s}{}^{\prime }\left(h-2a_{s}{}^{\prime }\right)+f_{y}A_{s}\left(h_{0}-a_{s}\right)}{2\left(f_{c}\cdot b\cdot \xi_{b}\cdot h_{0}+f_{y}{}^{\prime }A_{s}{}^{\prime }-f_{y}A_{s}\right)}$$

where *e*_0*b*_ is the limit eccentricity [48], *h* is the height of the section (mm), *b* is the width of the section (mm), *h*_0_ is the effective height of the section (mm), $\xi_{b}$ is the height of the relative limit compression zone, taking 0.52; $a_{s}{}^{\prime }$ is the distance from the resultant point of longitudinal reinforcement to the edge of the compression zone (mm), $a_{s}$ is the distance from the resultant point of longitudinal reinforcement in the tension zone to the tension edge (mm), $f_{y}$ is the design value of steel tensile strength (kPa), $f_{y}{}^{\prime }$ is the design value of steel compressive strength (kPa), $f_{c}$ is the design value of the compressive strength of concrete (kPa), $A_{s}$ is the cross-sectional area of tensile steel reinforcement (mm^2^) and $A_{s}{}^{\prime }$ is the cross-sectional area of the compressive steel reinforcement (mm^2^).

Eccentricity equation:

(6) $$e_{0}=\frac{1000M}{N}$$

where *e*_0_ is eccentricity (mm), *M* is the calculated value of the bending moment (kN·m), and *N* is the calculated value of the axial force (kN).

The judgment of large or small eccentric compression: if $e_{0}>e_{0b}$, it is large eccentric compression; if $e_{0}<e_{0b}$, it is small eccentric compression.

#### 3.2.2. Ultimate Bearing Capacity

Eccentricity amplification factor η, additional eccentricity $e_{a}$, and eccentricity $e_{i}$ and e are calculated according to the following equations:

(7) $$\eta =1+\frac{{\left(\frac{l_{0}}{h}\right)}^{2}\cdot \xi_{1}\cdot \xi_{2}\cdot h_{0}}{1400\cdot e_{i}}$$

(8) $$e_{i}=e_{0}+e_{a}$$

(9) $$e=\eta e_{i}+\frac{h}{2}-a_{s}$$

(10) $$l_{0}=0.54\cdot L$$

where the definitions and values of the coefficients $\xi_{1}$, $\xi_{2}$, and $e_{a}$ are the same as those in the code for the design of concrete structures [49], $e_{i}$ is the initial eccentricity (mm), $l_{0}$ is the calculated length (mm), and *L* is the arc length of the segment (mm).

The ultimate bearing capacity of the normal section is calculated according to the following equations:

(11) $$N_{cu}=\alpha_{1}\cdot f_{c}\cdot b\cdot x+f_{y}{}^{\prime }\cdot A_{s}{}^{\prime }-f_{y}\cdot A_{s}$$

(12) $$N_{cu}=\alpha_{1}\cdot f_{c}\cdot b\cdot x+f_{y}{}^{\prime }\cdot A_{s}{}^{\prime }-\sigma_{s}\cdot A_{s}$$

(13) $$N_{cu}e=\alpha_{1}\cdot f_{c}\cdot b\cdot x\cdot (h_{0}-\frac{x}{2})+f_{y}{}^{\prime }\cdot A_{s}{}^{\prime }\cdot (h_{0}-a_{s}{}^{\prime })$$

(14) $$\sigma_{s}=\frac{\beta -\xi }{\beta -\xi_{b}}f_{y}$$

where *N_cu_* is the ultimate bearing capacity of the normal section (kN), the definitions and values of $\alpha_{1}$ and β are the same as those in reference [49], ξ is the relative compression zone height, *x* is the height of the concrete compression zone (mm), and $\sigma_{s}$ is the stress of a tensile steel bar under small eccentric compression (kPa).

When it is determined to be large eccentric compression, the bearing capacity of the normal section is calculated using Equations (11) and (13). When it is determined to be small eccentric compression, the bearing capacity of the normal section is calculated using Equations (12)–(14).

## 4. Shell–Spring Model and Beam–Spring Model

### 4.1. Model Establishment

**Figure 6:**
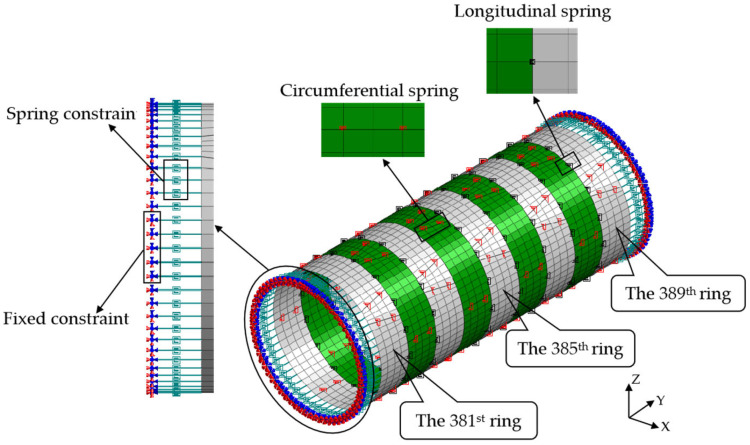
Shell–spring model.

**Figure 7:**
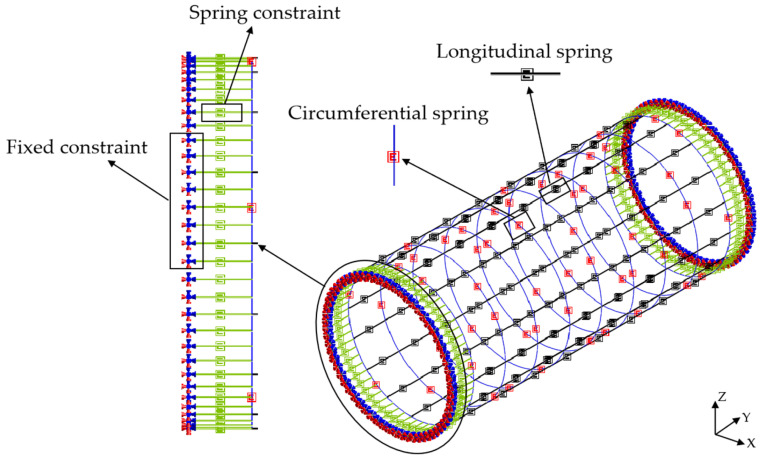
Beam–spring model.

To study the structure’s internal force and deformation of shield lining rings under surcharge loading, the shell–spring model and beam–spring model were used to simulate the 381~389 ring segments, respectively. The shell–spring model is shown in Figure 6, and the beam–spring model is shown in Figure 7.

### 4.2. Load Simulation

The ground heap soil height is set to 0 m, 5 m, and 10 m for the three calculation conditions. The load of the segments under different conditions is calculated using Equations (1)–(4), as shown in Table 2, where condition 3 is the load of the shield segment on site.

### 4.3. Simulation of Bolt Segments

The tunnel lining is composed of segments connected by bolts [50]. A spring is used to simulate the longitudinal and circumferential bolts between segments. To simulate the interaction between the edge ring segment and other tunnel rings, spring constraints are applied to the outer edges of the 381st and 389th ring segments. The spring can simulate axial tension and compression, radial shear, tangential shear, and rotational effects, and these are expressed as axial stiffness *k_x_*, radial shear stiffness *k_y_*, tangential shear stiffness *k_z_*, and rotational stiffness *k_rz_*, respectively. The mechanical parameters of the spring are shown in Table 3.

### 4.4. Shield Segment Simulation

The shell–spring model uses shell elements with four nodes and each node contains six degrees of freedom to simulate shield segments, and each ring segment is divided into 264 elements. The beam–spring model uses one-dimensional beam elements to simulate shield segments, and each ring segment is divided into 64 elements.

### 4.5. Contact Simulation between Segment and Stratum

**Figure 8:**
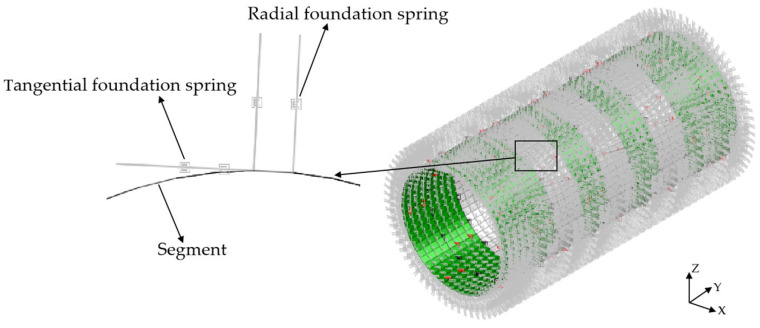
Shell–foundation spring model.

**Figure 9:**
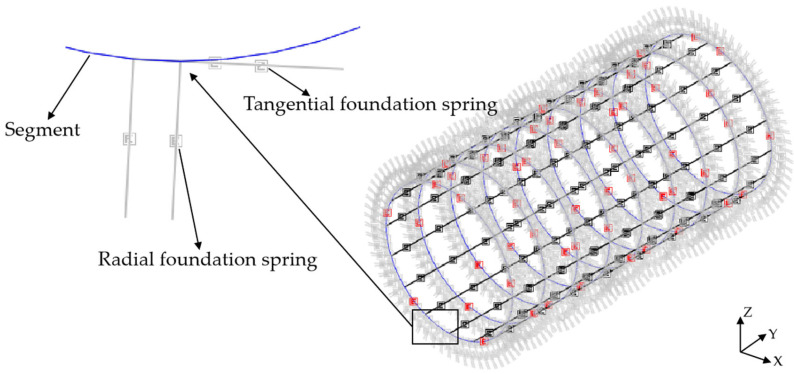
Beam–foundation spring model.

The “Winkler” foundation spring is used to simulate the interaction between the stratum and the segment structure, as shown in Figure 8 and Figure 9. The length of the foundation spring is set to 1 m, and the end of the spring is fixed. The interaction between the lining ring and stratum is divided into compression and friction. The radial foundation spring is used to simulate the compression of the stratum on the segment, which only reacts to pressure but not tension. When the radial spring is pulled, it will automatically fail and will not react to the lining ring. The tangential foundation spring is used to simulate the friction effect of strata on the segment, which can react to tension and pressure. According to the field geological conditions, the tangential foundation spring stiffness is 1/3 of the radial foundation spring stiffness.

## 5. Calculation Results and Comparative Analysis

### 5.1. Internal Force of Shield Segments

**Figure 10:**
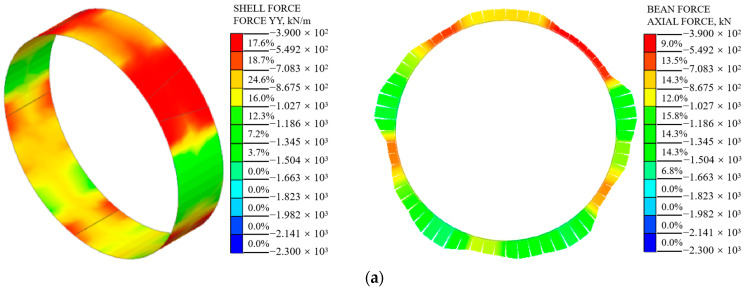

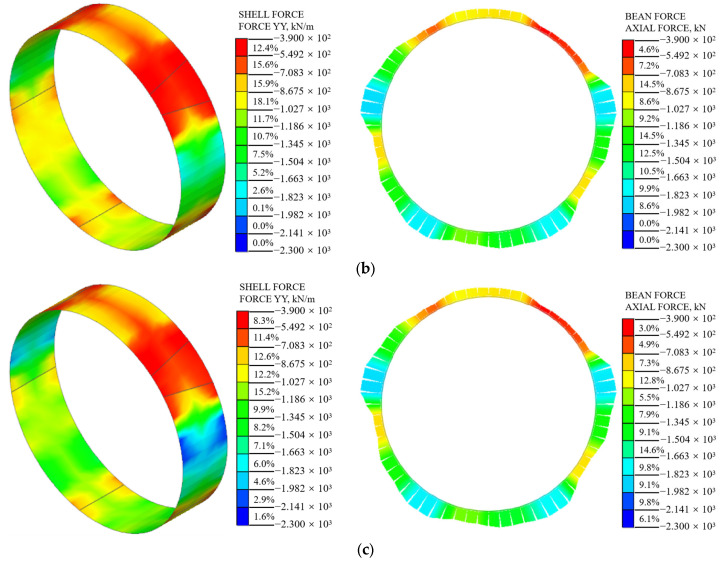
The axial force cloud diagram of the shell−spring model and the beam−spring model: (**a**) condition 1, (**b**) condition 2, and (**c**) condition 3.

**Figure 11:**
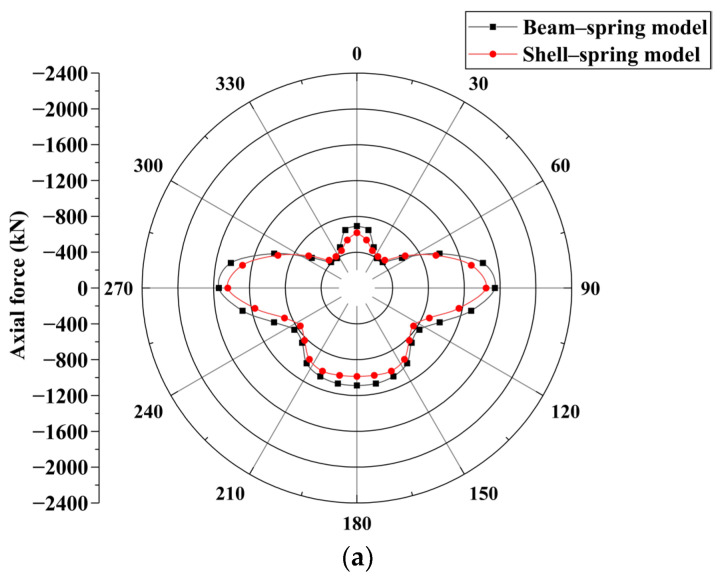

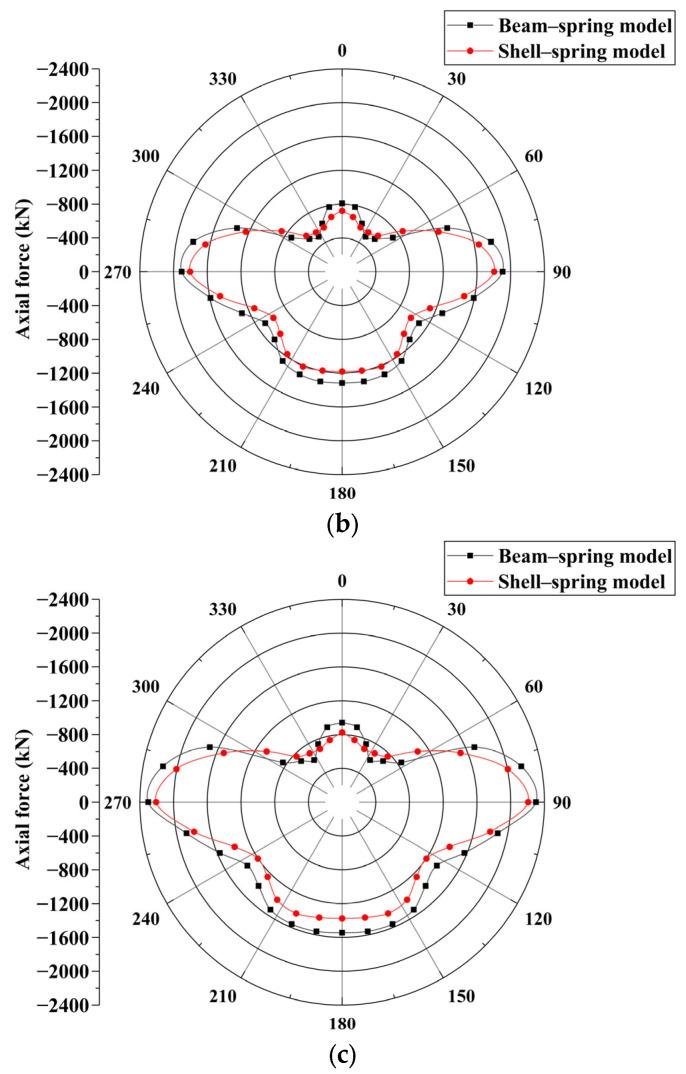
Comparison of axial force between shell–spring model and beam–spring model: (**a**) condition 1, (**b**) condition 2, and (**c**) condition 3.

**Figure 12:**
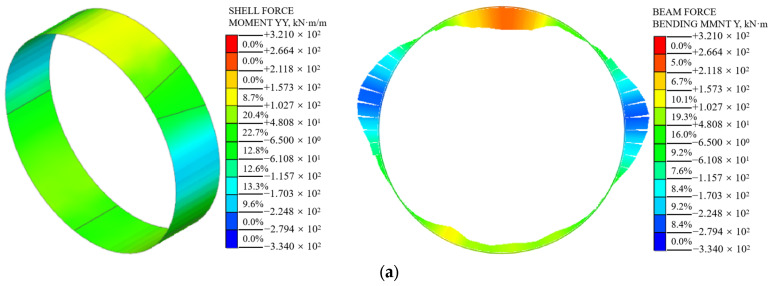

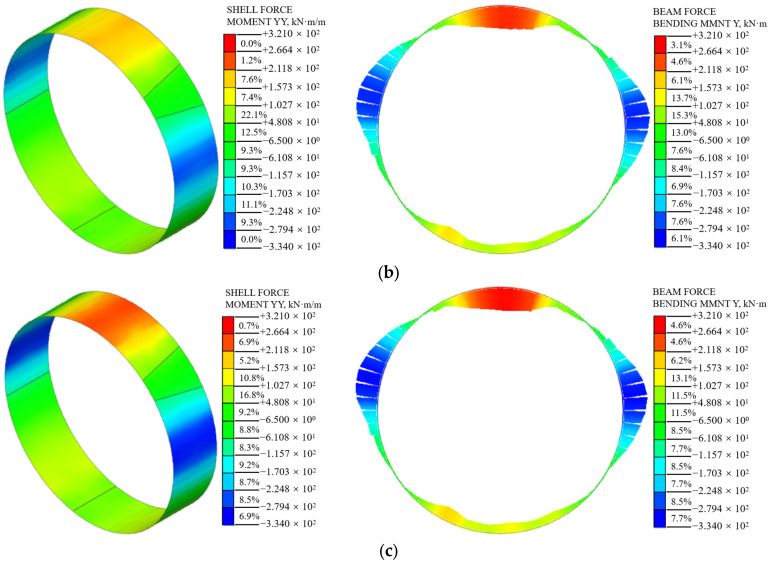
The bending moment cloud diagram of the shell–spring model and the beam–spring model: (**a**) condition 1, (**b**) condition 2, and (**c**) condition 3.

**Figure 13:**
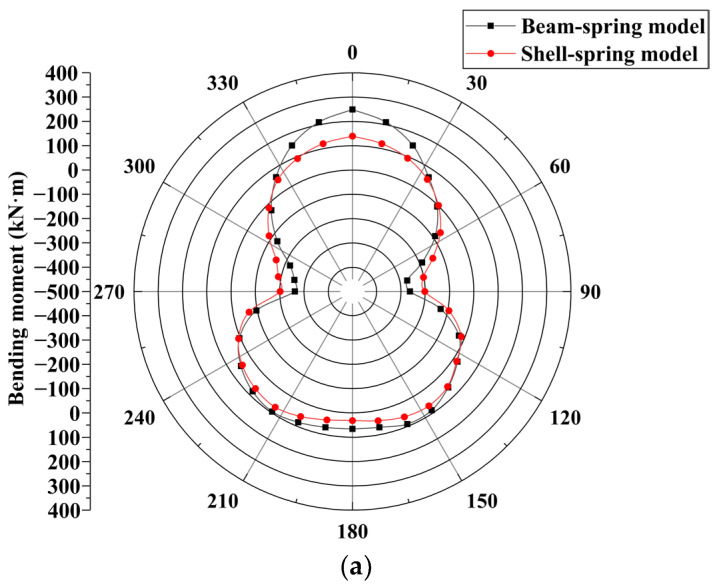

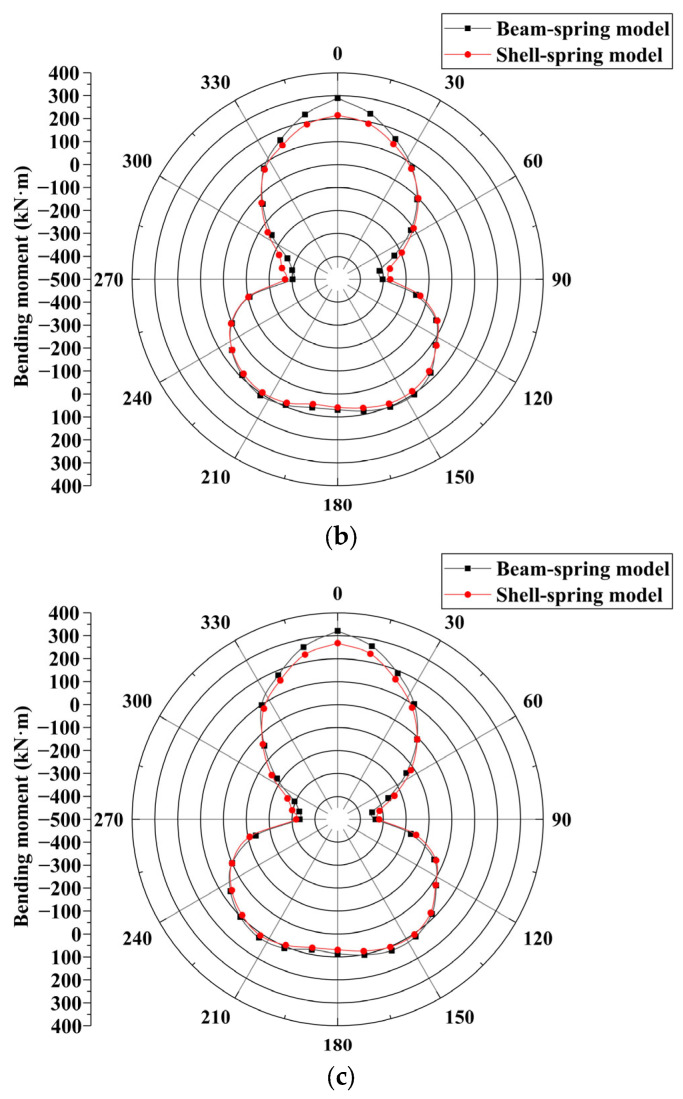
Comparison of bending moment between shell–spring model and beam–spring model: (**a**) condition 1, (**b**) condition 2, and (**c**) condition 3.

Under various loading conditions, the axial force and bending moment of the shield segment were calculated using the shell–spring model and the beam–spring model. The 385th ring segment was selected for analysis, and the internal force distribution cloud diagram and comparison diagram are presented in Figure 10, Figure 11, Figure 12 and Figure 13. The results indicate that, under different surcharge loading conditions, the internal force distribution of the segment structure obtained from both models is similar. The axial force reaches its maximum at the tunnel arch waist (90°, 270°). The segment structure experiences a maximum positive bending moment at the tunnel vault (0°) while the maximum negative bending moment occurs at the tunnel arch waist. As surcharge loading increases, the internal force of the segment structure also increases. In the shell–spring model, the internal force gradually decreases from the edges of the segment width toward the center. The shell–spring model provides a comprehensive representation of the segment’s mechanical response as it is a three-dimensional space model. It not only captures the transverse structural mechanics but also accurately reflects the longitudinal structural mechanics, thereby depicting the actual stress state of the segment. Significantly, the shield segment exhibits non-plane strain behavior, particularly at the joint spring connection, where notable stress concentration occurs. However, the beam–spring model employs a one-dimensional curved beam to simulate the segment structure. By default, each ring segment is assumed to be in a plane strain state. It fails to fully capture the true stress state when segments are assembled staggeredly.

#### 5.1.1. Axial Force

The axial force of the beam–spring model segment structure and the average axial force of the shell–spring model along the segment width direction were extracted to create a comparison graph, as shown in Figure 11. The axial force distribution of the segment structure calculated using the two models is similar, showing a butterfly shape. The axial force of the segment calculated using the shell–spring model is smaller than that calculated using the beam–spring model. In the beam–spring model, one-dimensional beam elements are used to simulate the shield segments, assuming them to be in a plane strain state. It is believed that the stress concentration phenomenon at the joint of the shield segment enhances the axial forces along the segment. However, in reality, the stress concentration at the joint only affects a small portion in the vicinity, resulting in localized reinforcement. Therefore, the beam–spring model tends to overestimate the stress concentration effect at the joint of the shield segment.

From condition 1 to condition 3, the maximum axial force appears at the arch waist, and the axial force of the segment is relatively small at the vault of the tunnel. The maximum axial force of the shell–spring model is 1443 kN, 1803 kN, and 2207 kN, respectively, and the maximum axial force of the beam–spring model is 1541 kN, 1904 kN, and 2300 kN respectively. The maximum axial force calculated using the shell–spring model is 6.4%, 5.3%, and 4.0% smaller than that of the beam–spring model, respectively. It can be observed that, as surcharge loading increases, the disparity in the computed axial forces between the two models gradually decreases. This is because, with an increase in external load, the stress concentration effects at the spring connections between the shield segments in the shell–spring model expand their influence range. This leads to strengthening along the longitudinal direction of the segments, causing the computed axial forces from both models to approach each other.

Overall, the disparity in the computed axial forces of the shield segments between the two models is not significant.

#### 5.1.2. Bending Moment

A comparison between the bending moment of the shell–spring model and the beam–spring model is shown in Figure 13. The bending moment distribution of the segment structure calculated using the two models is similar, forming a peanut-like shape. The bending moment of the segment calculated using the shell–spring model is smaller than that calculated using the beam–spring model. From condition 1 to condition 3, the maximum positive bending moment of the shell–spring model is 139 kN·m, 215 kN·m, and 268 kN·m, respectively, and the maximum negative bending moment is 202 kN·m, 270 kN·m and 318 kN·m, respectively. The maximum positive bending moment of the beam–spring model is 249 kN·m, 290 kN·m, and 321 kN·m, respectively, and the maximum negative bending moment is 263 kN·m, 303 kN·m, and 334 kN·m, respectively. The maximum positive bending moment calculated using the shell–spring model is 44.2%, 25.9%, and 16.5% smaller than that of the beam–spring model, respectively. The maximum negative bending moment calculated using the shell–spring model is 23.2%, 10.9%, and 4.8% smaller than that of the beam–spring model, respectively. The difference between the maximum positive bending moment (at the tunnel vault) of the segment structure calculated using the two models is larger than the maximum negative bending moment (at the tunnel arch waist). There is a significant difference between the tunnel vault bending moments calculated using the two models because the radial foundation spring of the tunnel vault is in a tensile state, which will not limit the deformation of the tunnel top segment structure. However, the radial foundation spring of the tunnel arch waist is in compression, which limits the deformation of this section of the structure. Therefore, the difference between the two bending moments calculated at the tunnel arch waist is relatively small.

Compared to the beam–spring model, the shell–spring model calculates a smaller internal force for the shield segment structure. This is consistent with the calculation results obtained in [26]. The beam–spring model utilizes one-dimensional beam elements to simulate the shield segments, assuming a default plane strain state for the segments. It assumes that the stress concentration at the spring connection enhances the longitudinal internal force of the segment. However, the actual stress concentration at the spring connection only affects a small region, resulting in local reinforcement. On the other hand, the shell–spring model is a three-dimensional spatial model that accurately represents the actual stress distribution and the local strengthening effect that occurs at the joint of the shield segment. The difference in axial force calculated using the two models is small, and the difference in bending moment is large. However, with an increase in surcharge loading, the difference in internal forces calculated using the two models gradually diminishes. This can be attributed to the expanding stress concentration range at the spring connection in the shell–spring model under higher external loads, thereby reinforcing the longitudinal internal force of the segment.

### 5.2. Comparative Analysis of Measured Deformation and Simulated Calculation of Shield Segment

**Figure 14:**
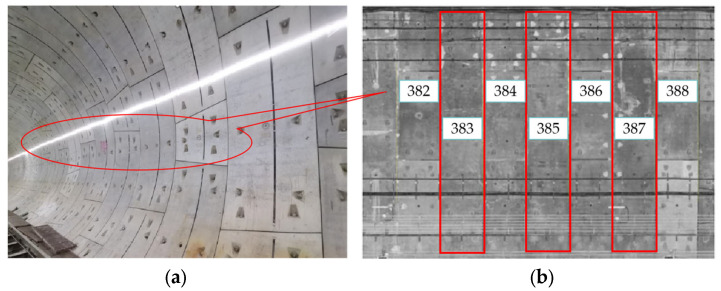
Shield tunnel segment: (**a**) measurement area and (**b**) scanning diagram.

The precise fast dynamic detection trolley is employed to perform a dynamic three-dimensional scanning of the shield tunnel segment while being subjected to onsite surcharge loading. The scanning positions of the shield segments are illustrated in Figure 14.

The red boxes in Figure 14 represent the measured segment rings. The horizontal convergence value, vertical convergence value, and ellipticity data of the 383rd, 385th, and 387th ring segments were collected and compared with the simulation data of condition 3 (the actual site condition). The comparative analysis results are shown in Table 4. The ellipticity of the lining ring of the subway tunnel does not exceed 6‰; therefore, the measured value and the calculated value meet the requirements of the specification. It can be seen from Table 4 that the calculated value of the beam–spring model is larger. Because the beam–spring model uses the beam element to simulate the segment, it is considered that the local deformation of a certain point on the beam element affects the whole ring segment, which exaggerates the local deformation effect. The deformation of the segment calculated using the shell–spring model is closer to the measured value, which can accurately reflect the actual deformation of the segment. The comparison results show that it is more reasonable to use the shell–spring model to simulate the shield segment.

### 5.3. Shell–Spring Model Validation

**Figure 15:**
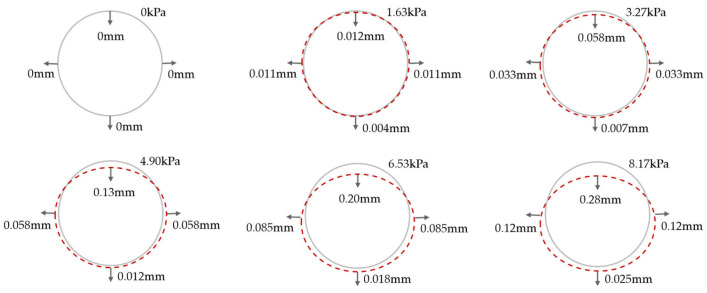
Convergent deformation of the middle ring.

The parameters of the shell–spring model established in this paper are adjusted by using the model test parameters provided in reference [12], and the deformation of the middle ring under different surcharge loading is calculated. The deformation calculation results of the middle ring are shown in Figure 15. The dashed line in Figure 15 represents the deformation of the segment ring, and the arrow points to the displacement direction, which is positive for downward and outward displacement. It can be seen that under the action of the surcharge loading above the shield tunnel, the middle ring is transversely elliptical, and the deformation of the arch waist on both sides is symmetrical. Specifically, the diameter decreases in the vertical direction and increases in the horizontal direction. With an increase in surcharge loading, the degree of transverse convergence deformation gradually increases. This is consistent with the trend of middle ring deformation described in [12].

**Figure 16:**
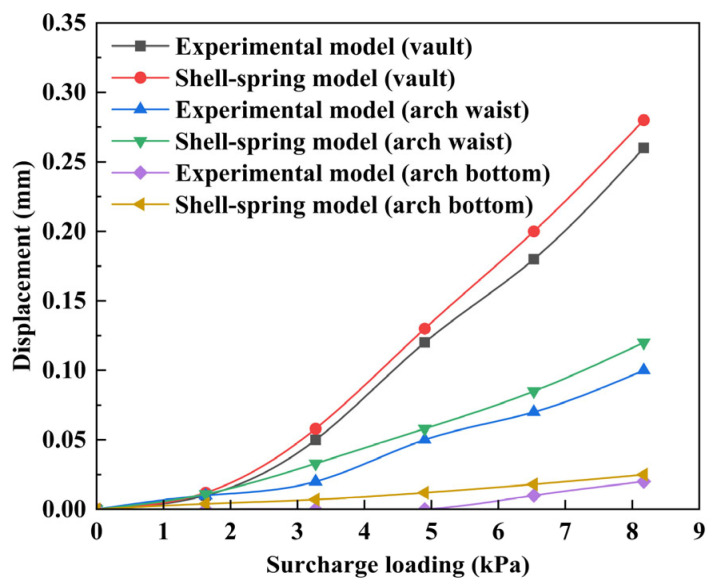
The displacement comparison results of the vault, arch waist, and arch bottom between the shell–spring model and the model test.

The displacement comparison results of the vault, arch waist, and arch bottom between the shell–spring model and the model test are shown in Figure 16. It can be seen from Figure 16 that the calculated value of the deformation of the middle ring is in good agreement with the experimental value, which verifies the accuracy of the shell–spring model. With an increase in surcharge loading, the increase rate of vault displacement is the fastest, the increase rate of arch waist displacement is lower than that of the vault displacement, and the change in arch bottom displacement is not apparent. Under the action of surcharge loading, the displacement relationship of the segment vault, arch waist, and arch bottom is vault > arch waist > arch bottom. Therefore, under surcharge loading, the monitoring of the displacement at the top of the shield tunnel should be strengthened.

### 5.4. Ultimate Bearing Capacity and Safety Evaluation of Segment Normal Section

The shell–spring model can simulate the stress and deformation of the shield segment more realistically. Therefore, the shell–spring model was selected to calculate the ultimate bearing capacity of the normal section of the 385th ring segment and evaluate its safety. Firstly, it is necessary to determine the most unfavorable normal section position of the shield tunnel. The determination method is as follows:

(15) $$e_{\max}=\max\left|\frac{1000M_{i}}{N_{i}}\right|$$

where *e*_max_ is the maximum eccentricity of the normal section of the shield segment (mm), *M_i_* is the calculated bending moment of the *i*-angle segment of the shield tunnel (kN·m), and *N_i_* is the calculated axial force of the *i*-angle segment of the shield tunnel (kN).

**Figure 17:**
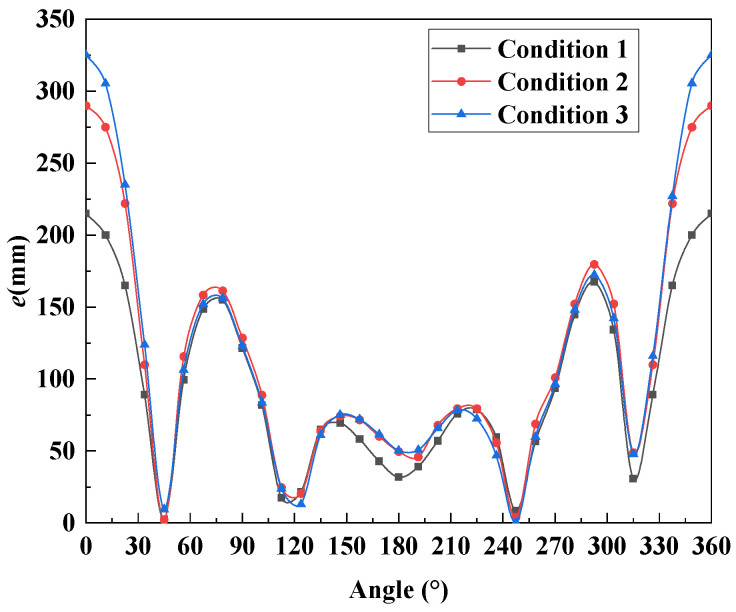
Eccentricity results from a normal section of shield tunnel segment with different angles.

According to Equation (15), the eccentricity results of the normal section of the shield tunnel at different angles are shown in Figure 17. The most unfavorable normal section of the shield tunnel is at the top of the tunnel (0°). Therefore, the ultimate bearing capacity calculation and safety evaluation analysis were carried out at the top of the tunnel.

According to Equations (5)–(14), the results of the ultimate bearing capacity of the normal section of the tunnel top segment are shown in Table 5. With an increase in surcharge loading, the ultimate bearing capacity of the shield segment gradually decreases. Compared to condition 1, condition 2 is reduced by 229 kN (10.3%), and condition 3 is reduced by 505 kN (22.7%).

The calculation equation of the safety factor of a normal section of the segment is as follows:

(16) $$S=N_{cu}/\gamma_{0}\gamma_{R}N$$

where $\gamma_{0}$ is the structural importance coefficient, taking 1.1, and $\gamma_{R}$ is the resistance partial coefficient, taking 1.35. The evaluation standard of the normal section safety of the segment is shown in Table 6.

**Figure 18:**
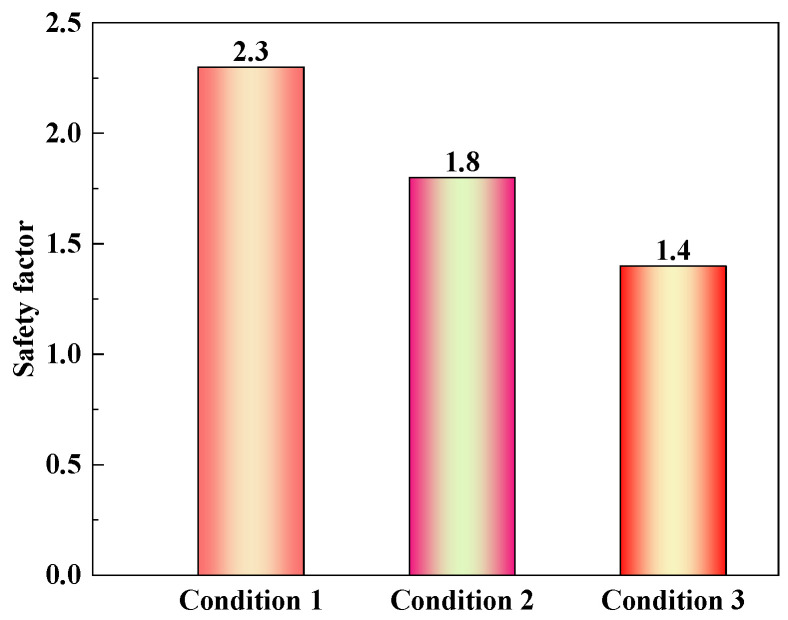
Histogram of safety evaluation coefficient of the shield tunnel.

Under different surcharge loading conditions, the safety evaluation coefficients of the shield tunnel are shown in Figure 18. With an increase in surcharge loading, the safety factor of the shield segment decreases. From condition 1 to condition 3, the safety factors of the shield segments are 2.3, 1.8, and 1.4, respectively. Compared with condition 1, condition 2 is reduced by 0.5 (21.7%), and condition 3 is reduced by 0.9 (39.1%). According to the evaluation standard of the normal section safety of segments in Table 6, the safety rating of the shield segment is *A_s_* under three conditions. The service state of the shield segment is normal, which meets the requirements of the subsequent normal use of the shield segment.

## 6. Conclusions and Recommendations

Based on a subway shield tunnel project in Zhengzhou city, the shell–spring model and beam–spring model were established using the geotechnical and tunneling software. The differences in the internal force and deformation of the two models under different surcharge loading were compared and analyzed. The shell–spring model was selected to calculate the ultimate bearing capacity of the normal section of the shield segment under different surcharge loading, and a safety evaluation was carried out. The main research and suggestions are as follows:

(1) With an increase in surcharge loading, the internal force of the segment structure increases gradually. In the shell–spring model, the internal force of the segment decreases gradually from the edge to the center of the segment width. The shield segment presents an obvious non-plane strain state. Especially at the joint spring connection, it shows obvious stress concentration. This is in good agreement with the actual stress state under the staggered assembly of shield segments;
(2) The internal force calculation results of the beam–spring model and the shell–spring model were compared and analyzed. The internal force of the beam–spring model is greater than that of the shell–spring model. The difference in axial force calculated using the two models is small, and the difference in bending moment is large. Nevertheless, with an increase in surcharge loading, the internal force difference of the segment structure calculated using the two models decreases gradually;
(3) The difference between the two models and the measured tunnel deformation was analyzed. The results show that the calculation results of the beam–spring model were larger than those of the shell–spring model and field measurement results. The calculated results of the shell–spring model are close to the measured values in the field, indicating that the shell–spring model truly reflects the actual deformation of the shield segment. The shell–spring model was used to calculate the segment ring deformation of the shield tunnel model test, and the calculated value agrees with the test value, which further verifies the accuracy and reliability of the shell–spring model. Therefore, it is more reasonable to use the shell–spring model to calculate the mechanical response of the segment structure. The shell–spring model should be selected for the mechanical response of the shield segment compared to the beam–spring model;
(4) Based on the conclusion (3), the shell–spring model is used to evaluate the safety of the shield segment. According to the eccentricity calculated using the shell–spring model, the most unfavorable normal section of the shield tunnel is located at the top of the tunnel. Therefore, under surcharge loading, the monitoring of the displacement at the top of the shield tunnel should be strengthened. When the displacement changes too much, the steel ring support is adopted in time to ensure the safety of the tunnel structure. With an increase in surcharge loading, the safety of the shield tunnel decreases gradually. Under the tunnel burial depth and stratum conditions described in this paper, the height of the piled soil above the shield tunnel should be controlled below 10 m. Surcharge loading above the shield tunnel should be reasonably controlled to meet the requirements of the normal use of a shield segment.

## Figures and Tables

**Table 1:** Physical and mechanical parameters of each soil layer.

Name of Soil Layer	Soil Thickness (m)	Unit Weight (kN/m^3^)	Internal Friction Angle (°)	Cohesion (kPa)	Poisson Ratio
⑧_5_ Clay silt	5.0	19.3	15	23.2	0.34
⑧_11_ Clay silt	4.8	19.5	16	18.1	0.38
⑧_12_ Silty clay	7.2	19.6	16	18.2	0.38
⑧_13_ Fine sand	11.0	19.8	18	18.6	0.38
⑧_14_ Clay silt	19.0	19.8	22	24.3	0.35

**Table 2:** Load calculation values.

Condition No.	Ground Heap Soil Height (m)	Vertical Earth Pressure (kPa)	Top Lateral Earth Pressure (kPa)	Bottom Lateral Earth Pressure (kPa)
1	0	258.9	116.5	167.1
2	5	305.7	137.6	188.1
3	10	352.5	158.6	209.2

**Table 3:** Mechanical parameters of the spring.

*k*_*x*_	*k*_*y*_	*k*_*z*_	*k*_*rz*_	
Axial tension and compression	Radial shear	Tangential shear	Positive rotation	Negative rotation
(10^8^ kN/m)	(10^6^ kN/m)	(10^6^ kN/m)	(10^5^ kN·m/rad)	(10^5^ kN·m/rad)
8.25	3.5	3.5	3.0	2.0

**Table 4:** Measured and simulated values of the deformation of the 383rd, 385th, and 387th ring segments.

Segment Ring	Condition No.	Horizontal Convergence Value (mm)	Vertical Convergence Value (mm)	Ellipticity (‰)
383rd	Measured value	13.7	18.8	5.24
	Shell–spring model	13.8	19.0	5.29
	Beam–spring model	14.8	20.2	5.65
385th	Measured value	13.8	19.0	5.29
	Shell–spring model	14.1	19.2	5.37
	Beam–spring model	15.1	20.3	5.71
387th	Measured value	13.6	18.6	5.19
	Shell–spring model	13.8	18.7	5.24
	Beam–spring model	15.0	20.2	5.68

**Table 5:** Calculation results of the ultimate bearing capacity of a normal section of tunnel top segment.

Condition No.	Bending Moment (kN·m)	Axial Force (kN)	Ultimate Bearing Capacity (kN)
1	139	650	2220
2	215	745	1991
3	268	825	1715

**Table 6:** The evaluation standard of normal section safety of a segment.

Safety Grade	Follow-Up Use Requirements	Service Condition
*A_s_*	S≥1.0	Normal
*B_s_*	1.0>S≥0.90	Degeneration
*C_s_*	0.90>S≥0.85	Pauperization
*D_s_*	S<0.85	Deterioration

## Data Availability

The data used to support the findings of this study are included within the article.
